# Gonadectomy effects on the risk of immune disorders in the dog: a retrospective study

**DOI:** 10.1186/s12917-016-0911-5

**Published:** 2016-12-08

**Authors:** Crystal R. Sundburg, Janelle M. Belanger, Danika L. Bannasch, Thomas R. Famula, Anita M. Oberbauer

**Affiliations:** 1 Department of Animal Science, University of California, One Shields Ave, Davis, CA 95616 USA; 2 Department of Population Health & Reproduction, School of Veterinary Medicine, University of California, Davis, CA 95616 USA

**Keywords:** Neuter, Dog, Immune function, Gonadectomy

## Abstract

**Background:**

Gonadectomy is one of the most common procedures performed on dogs in the United States. Neutering has been shown to reduce the risk for some diseases although recent reports suggest increased prevalence for structural disorders and some neoplasias. The relation between neuter status and autoimmune diseases has not been explored. This study evaluated the prevalence and risk of atopic dermatitis (ATOP), autoimmune hemolytic anemia (AIHA), canine myasthenia gravis (CMG), colitis (COL), hypoadrenocorticism (ADD), hypothyroidism (HYPO), immune-mediated polyarthritis (IMPA), immune-mediated thrombocytopenia (ITP), inflammatory bowel disease (IBD), lupus erythematosus (LUP), and pemphigus complex (PEMC), for intact females, intact males, neutered females, and neutered males. Pyometra (PYO) was evaluated as a control condition.

**Results:**

Patient records (90,090) from the William R. Pritchard Veterinary Medical Teaching Hospital at the University of California, Davis from 1995 to 2010 were analyzed in order to determine the risk of immune-mediated disease relative to neuter status in dogs. Neutered dogs had a significantly greater risk of ATOP, AIHA, ADD, HYPO, ITP, and IBD than intact dogs with neutered females being at greater risk than neutered males for all but AIHA and ADD. Neutered females, but not males, had a significantly greater risk of LUP than intact females. Pyometra was a greater risk for intact females.

**Conclusions:**

The data underscore the importance of sex steroids on immune function emphasizing a role of these hormones on tissue self-recognition. Neutering is critically important for population control, reduction of reproductive disorders, and offers convenience for owners. Despite these advantages, the analyses of the present study suggest that neutering is associated with increased risk for certain autoimmune disorders and underscore the need for owners to consult with their veterinary practitioner prior to neutering to evaluate possible benefits and risks associated with such a procedure.

## Background

Gonadectomy, commonly referred to as spay or neuter or castration, consists of the removal of reproductive organs and is commonly employed for pet population control and to confer health and behavior benefits [1–7]. Controlling the canine population has become an important issue for the United States, with the American Veterinary Medical Association identifying it as a societal welfare concern [3]. In the U.S., dogs are generally neutered after the age of 6 months and 64% of dogs are neutered [3]. In contrast to the U.S., some countries view neutering as an unnecessary or undesirable procedure. Sweden has a 98.9% intact rate for dogs [8] and other regions in Europe and South America also have high frequency of intact dogs [9–11]. Despite being one of the most common procedures performed by veterinarians in the United States, recent reports outline detrimental health impacts of neutering in the dog [1, 2, 12–16].

Neutering dogs has been shown to reduce the risk of certain reproductive problems such as mammary cancer [2], pyometra [1], or testicular cancer [2]. Recent publications have described health impairments caused by neutering with the majority of these studies focused on cancer and orthopedic conditions [1, 2, 13–16], with only a few studies assessing metabolic impacts. For example, neutered dogs have a decreased energy requirement and may become obese [12], and young dogs with immature thermoregulation, hepatic, renal, and protein binding function may be sensitive to the neutering surgical procedure [1, 13]. Skeletal development is regulated by gonadal hormones signaling closure of the physes of long bones; neutering prior to closure can result in elongated long bones [17] potentially contributing to joint disorders being more prevalent in dogs neutered before puberty [1, 2, 15, 16]. An increased prevalence of various neoplasms has also been correlated with gonadal function, as evidenced by the presence of gonadal hormone receptors in some neoplastic tissues [1, 14]. A threefold increase in transitional cell carcinoma of the urinary bladder for both sexes when neutered has been reported [2]. Moreover, hemangiosarcoma, osteosarcoma, and lymphosarcoma also show an increase in prevalence in neutered dogs compared to intact dogs [1, 2, 14–16]. Neutering is also associated with increased life expectancy, however compared to intact dogs, neutered dogs have an increased risk of death more from cancer and immune-mediated diseases and less from infections, trauma, and vascular disease [18].

One area that has not been explored in great depth is the impact of neutering on the immune system and its ability to combat disease. An association with neuter status has been reported for myasthenia gravis [19, 20] and immune-mediated hemolytic anemia [21] but little exploration for other immune mediated conditions. The reproductive system and the immune system are highly interdependent. The present study sought to evaluate if there was an association between neuter status and diseases thought to be modulated by the immune system. Immune tissues, such as thymus, thymocytes, lymph nodes and spleen, display gonadal steroid receptors [22, 23]. Ablation of the thymus, the tissue in which T lymphocytes mature, disrupts gonadal development, reduces sex steroid production, and interrupts reproductive cycles [22] whereas male mice neutered at an early age show delayed thymic involution, thymic hyperplasia and depressed humoral and cell-mediated immune function when given testosterone [24]. An ancillary question was whether males differed from females in the effects of neutering because it is known that there are distinct sex differences in immune components with female dogs displaying stronger cell-mediated and humoral responses, higher immunoglobulin levels [25, 26], greater percentages of CD8 T cells [27], and a greater susceptibility to autoimmune disorders when compared to males [24, 26, 27]. Other studies in mice demonstrate that females produce more immunoglobulins than males, mount a more vigorous immune cascade in response to infection or immunization, and generate more autoantibodies making females more susceptible to autoimmune disorders [23, 24, 28]. The role of estrogens in modulating immune function is particularly apparent within the female reproductive tract in which protection from infectious disease must be balanced with the acceptance of conceptus implantation [29]. The approach taken for the present study was a retrospective analysis of dogs seen at a veterinary teaching hospital for a range of autoimmune conditions affecting multiple body systems.

## Methods

The dogs under study were those seen by the Veterinary Medical Teaching Hospital at the University of California, Davis from January 1, 1995 through January 1, 2010. Medical records of 90,090 individual dogs, inclusive of all AKC breeds as well as mixed breeds, were examined. The process of examining the medical records was described in [30]. Briefly, patients were classified as affected by a disease only if a confirmed diagnosis based on symptoms and test results was indicated in the records. If a disease was only regarded as a possible suspect, but not confirmed, the patient was not designated as affected by the disease. All data fields within the medical records were analyzed by searching for key words and phrases of the diseases evaluated. Spayed female dogs and castrated male dogs are collectively referred to as neutered. Sex status was also recorded for each dog as either intact or neutered. The date of initial disease diagnosis was determined from the records. To ensure reliability, age of neuter was only determined for dogs neutered at the teaching hospital. Dogs that were neutered within 150 days of that initial diagnosis were classified as intact at the time of diagnosis.

The immune medical conditions selected were those having a prevalence in excess of 0.1% in the dog population studied, recognized as having an underlying immune causality, and impacting multiple organ systems: atopic dermatitis (ATOP) autoimmune hemolytic anemia (AIHA), canine myasthenia gravis (CMG) colitis (COL), hypoadrenocorticism (ADD), hypothyroidism (HYPO), immune-mediated polyarthritis (IMPA), immune-mediated thrombocytopenia (ITP), inflammatory bowel disease (IBD), lupus erythematosus (LUP), and pemphigus complex (PEMC) [31–37]. Lupus erythematosus included diagnoses of both systemic lupus erythematosus (SLE) and discoid lupus erythematosus (DLE) based upon the view that DLE is a localized presentation of SLE [32]. Pyometra (PYO) was used as a positive control; pyometras recorded for neutered females were stump pyometras.

Binary data were used to determine prevalence of disease for the overall population and categories differentiated by parameters of sex and age at neuter. Prevalence was calculated by dividing the total number of dogs with disease within that category by the total number of dogs in that category.

Accordingly, for a given disease, the information available is simply a set of counts of the number of cases and controls categorized into one of four sex classes (F = intact females, NF = neutered females, M = intact males and NM = neutered males). Evaluation of the differences in the probability of disease by sex class was straightforward, taking advantage of the binomial density. That is, for a given disease and a given sex class *i* (*i* = F, NF, M, NM), n_cases i_ ~ Binomial(n_cases i_ + n_controls i_ , p_i_) where n_cases i_ was the number of observed cases in sex group *i*, n_controls i_ was the number of unaffected dogs in sex group *i* and p_i_ was the probability of disease in sex group *i*. We estimated p_i_ for each disease along with associated values of relative risk (RR) and the odds ratio (OR) across the sex groups [38].

Specifically, we evaluated the relative risk for females as RR_F_ = p_NF_/p_F_ and that for males as RR_M_ = p_NM_/p_M_. In addition, we defined the odds ratios for females and males as OR_F_ = [p_NF_/(1-p_NF_)]/[p_F_/(1-p_F_)] and OR_M_ = [p_NM_/(1- p_NM_)]/[p_M_/(1-p_M_)], respectively. Estimation of these measures of differences in disease prevalence across the sex classes, along with their associated confidence intervals, can be accomplished by several methods [38] and we opted to use a hierarchical Bayesian framework facilitated through the public-domain software Stan [39] which can be accessed through the public-domain language R [40]. Both the OR and the RR are presented. Though different in their interpretation, and more importantly in their calculation, these two statistics of disease risk are expected to be nearly identical when a disease is of low prevalence. Slight variations between the two values may exist for conditions having higher prevalence with the OR expected to inflate the perceived risk [41].

A hierarchical model was used because many of the diseases to be evaluated are uncommon. Accordingly, estimating p_i_, and perhaps more importantly the standard error of p, in those cells was expected to be quite imprecise. Using a hierarchical model allows imposition of structure on the estimates of p_i_, thereby providing for more reasonable and stable estimates of p_i_ and the credible interval around p_i_ (and thus the RR and OR) [42]. Estimation of p_i_ was accomplished through the log-odds, i.e., log(p_i_ /(1- p_i_)) = sex_i_ (*i* = F, NF, M, NM) where sex_i_ was the sex group effect, which, in our hierarchical Bayesian model had a prior density of sex_i_ ~ N(0, σ^2^). The next step in this hierarchical model, intending to address the challenge of evaluating uncommon disorders, we considered the weakly informative prior of a half-Cauchy for σ [43], that is σ ~ Cauchy(0,25). In fitting of this hierarchical model, the MCMC (Monte Carlo Markov Chain) sampling process was run in 4 chains, where each chain was based upon 20,000 total samples, and the first 5000 were removed as part of the warm-up process, subsequently thinned to every 20-th sample, resulting in a MCMC sample of 3000 values [39]. Convergence to the posterior density was evaluated by the Gelman-Rubin test statistic, where values less than 1.05 would indicate that the MCMC sampling process was adequate to the data evaluated [44].

## Results

**Fig. 1:**
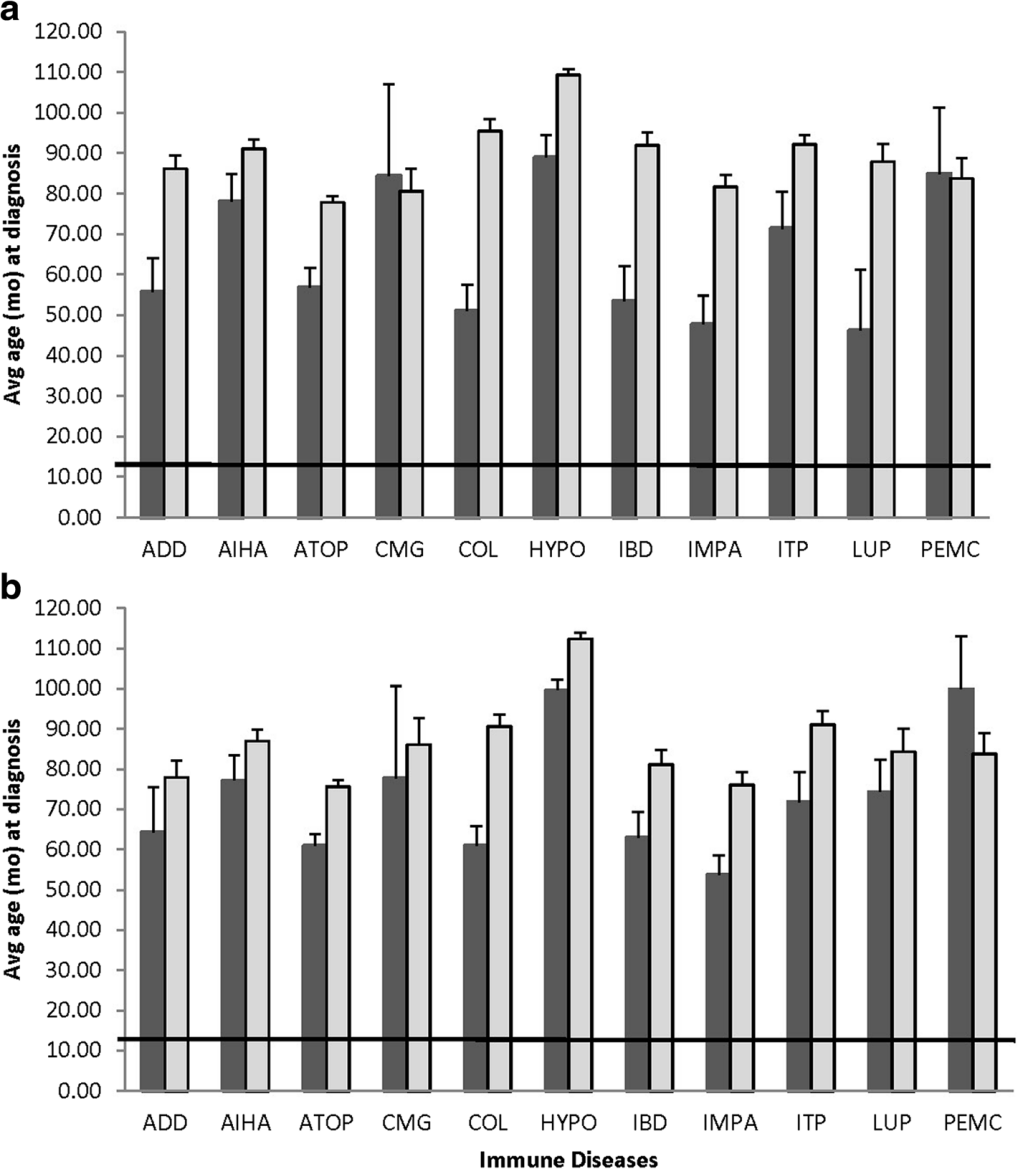
Age at disease diagnosis (mean and standard error of the mean) relative to sex category for females (**a**) and males (**b**). The solid horizontal line indicates the median age at neuter

The population demographics are presented in Tables 1 and 2. Neutered dogs made up 75.93% of the dogs evaluated. The overall proportion of males and females in the population under study with these immune conditions was 5.2 and 5.5%, respectively. The proportion of dogs with these conditions decreased over time with an aggregate prevalence of 7.55% in 1995 and 5.53% in the final year evaluated. There were 6281 dogs neutered at the teaching hospital with an average age (± standard error) at neuter of 32.36 ± 0.73 and 28.61 ± 0.55 months for males and females, respectively. The median age of neuter (± standard error) was 13.40 ± 0.91 and 13.08 ± 0.69 months for males and females, respectively. Average age of diagnosis for each condition is presented in Fig. 1. For the cases in which age at neuter could be assessed, there were no differences in age of gonadectomy as a function of disease (*p* > 0.1). The data were not extensive enough to assess the impact of early or late neutering on condition expression. In general, the diagnosis of disease for intact dogs occurred at younger ages than neutered dogs. Diagnosis of these diseases for neutered dogs occurred more than 20 months post-gonadectomy.

**Table 1:** Profile of dog population evaluated

Sex	Number of dogs	% of total
Intact Female	9133	10.14
Neutered Female	36574	40.60
Intact Male	12555	13.94
Neutered Male	31828	35.33

**Table 2:** Disease distribution by sex class

Disease	Intact females	Neutered females	Intact males	Neutered males	Percent in study population
Atopic Dermatitis (ATOP)	83	745	169	641	1.82
Autoimmune Hemolytic Anemia (AIHA)	38	256	38	176	0.56
Canine Myasthenia Gravis (CMG)	11	49	6	38	0.12
Colitis (COL)	61	267	109	256	0.77
Hypoadrenocorticism (ADD)	25	147	20	113	0.34
Hypothyroidism (HYPO)	62	750	210	678	1.89
Immune-Mediated Polyarthritis (IMPA)	24	170	56	141	0.43
Immune-Mediated Thrombocytopenia (ITP)	21	262	29	151	0.51
Inflammatory Bowel Disease (IBD)	20	189	46	167	0.47
Lupus Erythematosus (LUP)	6	74	30	47	0.17
Pemphigus Complex (PEMC)	13	71	11	55	0.17
Pyometra (PYO)	176	27	NA	NA	0.44

**Fig. 2:**
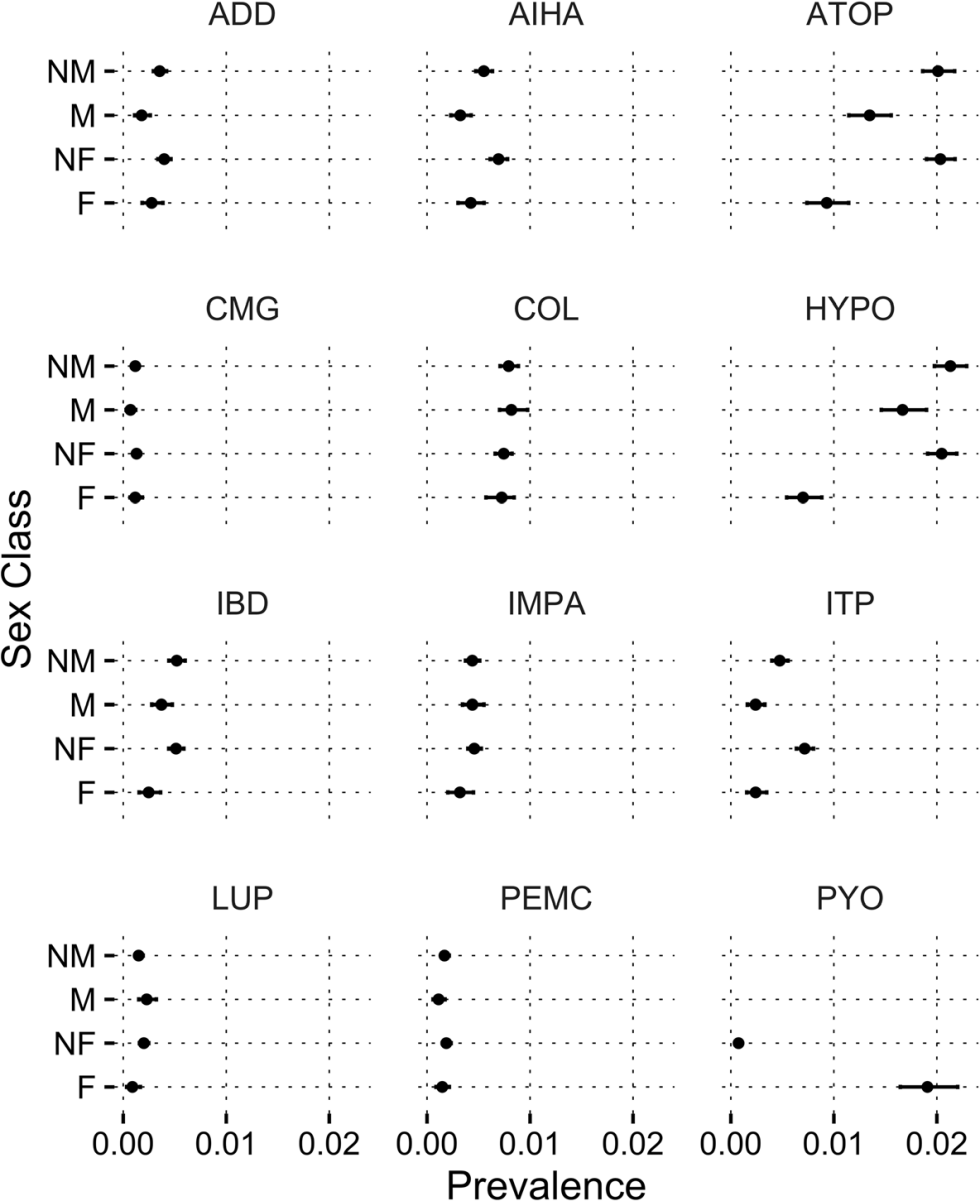
Caterpillar plot of the predicted probability that a dog would have each of the ten immune conditions based on neuter category parameters (data presented as mean probability ± 95% confidence interval)

The predicted probability of disease prevalence and the 95% confidence interval (CI) for each condition are depicted in Fig. 2. Overall, predicted probability for disease prevalence was equivalent between intact males and intact females with the exception of atopic dermatitis which was less prevalent in females than males. Prevalence of CMG, COL, IMPA, LUP, and PEMC was statistically equivalent across all four sex categories.

Table 3 lists the ORs and RRs for each condition. In general, the OR and RR for each condition were nearly identical. Neutering increased prevalence and risk of expressing the disease over that observed for the intact dog for some of the conditions assessed. Specifically, being neutered increased the risk for both males and females for ADD, AIHA, ATOP, HYPO, IBD, and ITP. An increased neutering risk for LUP was only observed for females. In the conditions of ATOP, HYPO, IBD, and ITP in which both neutered males and females exhibited greater risk of disease, neutered females had an approximately 1.5 to 2 times higher risk than that for neutered males. Hypoadrenocorticism (ADD) showed a greater risk for neutered males than neutered females. No conditions exhibited a reduced risk with neutering except, as expected, pyometra was significantly more likely to occur in intact females.

**Table 3:** Odds ratios (OR) and relative risk (RR) (± standard error) for the neutered female and male being more likely to express the condition (NA is not applicable)

Disease	OR		RR	
	Neutered female	Neutered male	Neutered female	Neutered male
Atopic Dermatitis (ATOP)	2.24 ± 0.27*	1.51 ± 0.27*	2.21 ± 0.26*	1.50 ± 0.13*
Autoimmune Hemolytic Anemia (AIHA)	1.67 ± 0.28*	1.76 ± 0.31*	1.67 ± 0.28*	1.76 ± 0.31*
Canine Myasthenia Gravis (CMG)	1.19 ± 0.37	1.97 ± 1.01	1.19 ± 0.37	1.97 ± 1.01
Colitis (COL)	1.03 ± 0.11	0.98 ± 0.09	1.03 ± 0.11	0.98 ± 0.08
Hypoadrenocorticism (ADD)	1.49 ± 0.32*	2.07 ± 0.54*	1.49 ± 0.32*	2.07 ± 0.53*
Hypothyroidism (HYPO)	3.03 ± 0.39*	1.29 ± 0.11*	2.99 ± 0.39*	1.28 ± 0.10*
Immune-Mediated Polyarthritis (IMPA)	1.49 ± 0.37	1.02 ± 0.14	1.49 ± 0.37	1.02 ± 0.14
Immune-Mediated Thrombocytopenia (ITP)	3.14 ± 0.73*	2.05 ± 0.42*	3.13 ± 0.73*	2.05 ± 0.42*
Inflammatory Bowel Disease (IBD)	2.20 ± 0.54*	1.43 ± 0.23*	2.19 ± 0.54*	1.43 ± 0.23*
Lupus Erythematosus (LUP)	2.64 ± 1.24*	0.68 ± 0.16	2.64 ± 1.24*	0.68 ± 0.16
Pemphigus Complex (PEMC)	1.35 ± 0.39	1.64 ± 0.56	1.35 ± 0.39	1.64 ± 0.56
Pyometra (PYO)	0.04 ± 0.01*	NA	0.04 ± 0.01*	NA

Asterisks indicate a significant difference from the intact counterpart (*p* < 0.05)

## Discussion

The objective of this study was to determine if prevalence and risk of canine diseases associated with immune function were affected by neutering. There have been many reports on the benefits of neutering [18] and although several studies have looked at the change in prevalence of various conditions in relation to neuter status [1, 15, 16, 45], there are few reports associating neuter status with immune disorders [19, 21]. For several diseases evaluated in the present study, neutered dogs showed higher prevalence than their intact counterparts and intact females had a reduced risk for most of the diseases with the clear exception of pyometra, a disorder specifically chosen to have greater incidence in intact females. For conditions other than pyometra, neutering did not reduce the risk of immune disease. Considering both female sex categories together, the proportion of females diagnosed with these immune conditions was slightly greater than when compared to the aggregate male data. A 1999 review by Pedersen reported the greatest prevalence of immune conditions occurred in intact females and the lowest with intact males [32] an assertion supported by the human literature [46] although those previous reports focused solely on intact individuals. In the canine literature, diagnoses of atopic dermatitis [47], CMG [20], IBD [48], lupus erythematosus [49], and PEMC [50] do not exhibit sex differences whereas for ADD, AIHA, IMPA, and ITP females have approximately a twofold higher risk than males [24, 51, 52]. A sex differential for those diseases did not show significant differences in the present study although there was a tendency of greater prevalence in females for AIHA, ADD, and ITP but not at a twofold higher risk as indicated in the literature possibly due to the different populations assessed in each study.

Hoffman et al. noted that neutered dogs had longer lifespans and were less vulnerable to death by infectious disease than intact dogs [18]. The authors postulated that gonadal steroid profiles [53] present in the intact dog act as immune-suppressants making the intact dog susceptible to infectious disease. Although gonadal steroids are known to impact immune responsivity as noted above, additional factors likely influence incidence and severity of disease [54]. The present study evaluated conditions that have a different etiology and progression than pathogenic infectious diseases; it is reasonable that the immune conditions evaluated in the present study would respond differently to gonadal steroids. Furthermore the current study looked only at disease diagnosis and expression but not mortality associated with disease.

Some conditions were diagnosed at earlier ages in the intact dogs, more frequently for females than males. Possible explanations for this observation include that the health status of intact dogs may be more heavily scrutinized prior to inclusion into breeding programs, hormonal cyclicity related to gestation or the estrous cycle [55], and an impact of a gravid uterus on expression of immune conditions. Despite that for some conditions intact females were diagnosed at earlier ages, intact females showed the lowest risk in many of the diseases assessed, suggesting a health benefit for not neutering female dogs. Females have higher immunoglobulin levels (reviews include [23, 24]) partly due to increased immunoglobulin production prompted by estrogen. In mice, estrogen increases immunoglobulin production and enhances antibody response, while testosterone inhibits humoral immunity [26]. Perhaps this permits a stronger humoral and cell-mediated immune response than seen in males and a stronger response may benefit intact female dogs by detecting threats more quickly and efficiently or be better able to distinguish between self and non-self.

However, in previous studies of other species, intact females also tend to be more prone to many autoimmune diseases. In the present study, females had a slight, though not significant, greater prevalence than males for autoimmune conditions. An increased prevalence in autoimmune diseases may reflect estrogens driving immunoglobulin production and/or permitting autoantibodies to reach maturity whereas testosterone, through its inhibitory action on immunoglobulin production, may prevent autoantibody production.

One might predict that ablation of estrogens would reduce the risk for these conditions; that hypothesis was not supported by the present data. Similarly, androgens have been found to be immunosuppressors [56]; neutering may release this inhibitory action and promote a more active immune response. Further evaluation is needed to assess whether a more robust immune response is a benefit or detriment with respect to risk for autoimmune disorders in the dog. Results from the present study, which did not show intact females to be at greater risk for autoimmune disorders, may be due to the particular autoimmune diseases assessed, the methodology of experiments (e.g. retrospective or case study), species evaluated, or whether the parameter of neuter status was included. Most studies that have demonstrated a preponderance of female autoimmune disease expression have focused upon intact animals.

In the few studies evaluating gonadectomy, neutering of male and female mice resulted in thymic and lymphoid tissue hyperplasia [57]. In normal development, the thymus begins to involute following puberty as a consequence of elevated gonadal steroids; the involution is presumably responsible for the age associated decline in immune function [58]. The withdrawal of the steroids by gonadectomy may reverse the involution although older animals may be less responsive [59]. Additionally, thymic hyperplasia has been associated with autoimmune conditions [60] suggesting gonadectomy may potentiate the expression of immune-mediated diseases. Ovariectomy in rats was shown to increase autoantibodies to thyroglobulin [61] and, in agreement with the present data, a study correlated neutering in dogs with hypothyroidism in both sexes [62] which was speculated to have been associated with autoantibody destruction of the thyroid gland although autoantibody levels were not assessed. Furthermore, gonadectomy in both sexes of mice potentiates Sjorgren’s syndrome, an autoimmune condition [63]. In contrast, canine diabetes mellitus is associated with pancreatitis or autoimmune destruction of the pancreas [64] and a study by Pöppl et al. demonstrated recovery from the condition upon neutering [65]. Taken together these findings suggest differential regulation of autoantibody production in response to gonadal steroids; this view is supported by human literature that both demonstrates differences in tissue specific response to steroids for females and males as well as differences in expression of autoimmune conditions [66].

In fact, for ADD, neutered males were at greater risk than intact males and in females neutering only minimally altered the risk of disease. The data indicate a protective role for androgens in ADD, a view supported by an early study of a female child with hypoadrenocorticism whose condition improved with the administration of androgens [67] and other human studies that indicate a correlation between hypogonadism and severity of the hypoadrenocortical condition most particularly evident in males [68]. Additionally, studies using mice have shown that gonadectomy affects the hypothalamic-pituitary-adrenal axis in a sex-specific manner: neutered males produce larger quantities of corticosterone from the adrenal [57]. That alteration may account for the higher risk for hypoadrenocorticism observed in the neutered male dogs along with a role for androgens in adrenal function. The finding also illustrates that specific tissues respond to the gonadal steroids differentially possibly accounting for the sex specific risks seen for immune diseases with neutering.

Lupus in mice has been found to be accelerated with estrogen administration and blunted with androgen administration [23, 24]. Interestingly in mice prone to systemic lupus erythematosus, neutering reduces mortality in female mice yet guarantees mortality in males; treatment of intact females with testosterone and dihydrotestosterone extends their lifespan [69]. Although in the present study, the risk of canine lupus erythematosus was higher in neutered females than either male category or intact females, caution is needed in interpreting this risk because the prevalence of lupus in intact females in the present study was very small and other studies in multiple species, including dogs, show a correlation between estrogen and increased morbidity of lupus [69].

For conditions with an elevated risk associated with neutering, the gonadal steroids may serve a protective role. Diseases such as atopic dermatitis and IBD, where the mechanism of disease onset involves inflammation, higher concentrations of sex steroids present in intact dogs may exert an anti-inflammatory effect to prevent disease progression similar to what has been reported in mice [70]. The anti-inflammatory effects of estradiol are believed to be mediated by estrogen receptor blocking intracellular trafficking of the p65 transcription factor, that would normally be translocated to the nucleus to drive transcription of proinflammatory proteins such as cytokines and chemokines [71]. A similar anti-inflammatory effect may occur in the dog. Additionally, estradiol has an antioxidant effect in mice [70] and a recent study in dogs reported that gonadectomized females have a greater risk for oxidative stress [72] which may lead to greater susceptibility for immune diseases.

Canine atopic dermatitis is a complex disease influenced by genetic predisposition and environmental factors. Nearly half of the risk for atopy is attributed to the genetic background of the dog, with as many as 54 different genes exhibiting differential transcription in atopy cases [73]. Environmental factors vary with atopy risk influenced by geographic location, rainfall, proximity to other animals, and rural versus urban habitat [73] though no published studies assessed the influence of sex steroids on atopy. It is a common disorder with estimates of up to 10% of dogs affected with both sexes equally impacted [47] and even though the present study population prevalence was only ~1.8%, it was one of the most prevalent diseases under study consistent with previous reports of conditions that prompt veterinary visits [74]. The lower prevalence may reflect the population under study representing patients at a referral hospital with only the most severe cases seen. Neutered females had a much greater risk than the other sex categories indicating a strong suppressive effect of estrogens on the expression of the disease. Atopic dermatitis involves the production of IgE or IgGd, a subset of IgG antibodies, in response to antigens in the environment with IgE being viewed as more diagnostic and implicated in causality though there exists conflicting reports on the latter [75–77].

Differences in sensitization of a biological pathway in erythrocytes, thrombocytes, or immune cells of the dog by estrogen or progesterone in the female may play a role in the hematologic diseases. AIHA and ITP involve the destruction of erythrocytes and thrombocytes, components of blood that have a high degree of regeneration. If estrogen or progesterone control cell surface markers on thrombocytes or erythrocytes, removal of estrogen or progesterone might change cell surface markers and cause the thrombocytes or erythrocytes to be recognized as non-self. A comparable hypothesis was proposed for colon cancer in women where it was postulated that estrogen sensitized and inhibited colon cells from becoming cancerous because following estrogen withdrawal, there was an increase in risk for having tumors [78]. Similar hypotheses have also been postulated for hemangiosarcoma and mast cell tumor development in the dog [1].

It is important to note that even though the study population included more than 90,000 dogs and expression of the diseases were statistically prevalent, the actual prevalence of the conditions studied was not high and the prevalence of diagnosis declined over the 15 years evaluated. Additionally, this was a retrospective study limited to the dogs seen at a referral veterinary hospital and may not reflect the prevalence of the population at large but rather may be biased to complex or more severe cases. However, the prevalence of disease seen in the present study does concur with other studies of much larger numbers of dogs, such as a study done using records from the Banfield hospital population which found 1.7% of dogs were diagnosed with atopy out of a population of 1,345,697 dogs in 2007 [79], a similar prevalence to that seen with the present study. Finally the study indicates a correlation of neutering and disease status but because of the retrospective nature does not prove causality. This is especially true given the known genetic contributions to some canine autoimmune disorders [80]. Thus, although the current results indicate a significant risk associated with neutering, additional prospective studies are warranted to confirm the relationship of sex steroids and autoimmune risk seen in this study.

## Conclusions

Neutering has tangible benefits such as eliminating unwanted reproduction in dogs [1, 81], lowering the incidence of reproductive cancers, and increasing lifespan [2, 18]. In contrast, neutered females may display increased dominance aggression and urinary incontinence [81–83] and susceptibility to certain neoplasias [14]. The role of neutering on immune function must also be taken into account. Six of the autoimmune diseases evaluated showed an increased prevalence in neutered dogs, supporting the concept that neutering may have a detrimental effect on a canine’s health and immune function. Lupus was the only disease that did not show increased prevalence in neutered dogs of both sexes compared to intact dogs. These findings indicate the importance of sex steroids on immune function and suggest a role of these hormones on self-recognition. Furthermore, the data underscore the importance of dog owners engaging in a thoughtful conversation with their veterinarian as to the advantages and potential risks of neutering.

## Abbreviations

ADD: Hypoadrenocorticism
AIHA: Autoimmune hemolytic anemia
ATOP: Atopic dermatitis
CMG: Canine myasthenia gravis
COL: Colitis
F: Intact female
HYPO: Hypothyroidism
IBD: Inflammatory bowel disease
IgE: Immunoglobulin E
IgG: Immunoglobulin G
IMPA: Immune-mediated polyarthritis
ITP: Immune-mediated thrombocytopenia
LUP: Lupus erythematosus
M: Intact male
NF: Neutered female
NM: Neutered male
OR: Odds ratio
PEMC: Pemphigus complex
PYO: Pyometra
RR: Relative risk

## References

[1] Kustritz R (2012). Effects of surgical sterilization on canine and feline health and on society. Reprod Domest Anim.

[2] Kustritz MVR (2007). Determining the optimal age for gonadectomy of dogs and cats. J Am Vet Med Assoc.

[3] Trevejo R, Yang M, Lund EM (2011). Epidemiology of surgical castration of dogs and cats in the United States. J Am Vet Med Assoc.

[4] Wright JC, Nesselrote MS (1987). Classification of behavior problems in dogs: distributions of age, breed, sex and reproductive status. Appl. Anim. Behav. Sci..

[5] Gershman KA, Sacks JJ, Wright JC (1994). Which dogs bite? A case–control study of risk factors. Pediatrics.

[6] Looney AL, Bohling MW, Bushby PA, Howe LM, Griffin B, Levy JK, Eddlestone SM, Weedon JR, Appel LD, Rigdon-Brestle YK (2008). The Association of Shelter Veterinarians veterinary medical care guidelines for spay-neuter programs. J Am Vet Med Assoc.

[7] Diesel G, Brodbelt D, Laurence C (2010). Survey of veterinary practice policies and opinions on neutering dogs. Vet Rec.

[8] Sallander M, Hedhammar A, Rundgren M, Lindberg J (2001). Demographic data of a population of insured Swedish dogs measured in a questionnaire study. Acta Vet Scand.

[9] Acosta-Jamett G, Cleaveland S, Cunningham A (2010). Demography of domestic dogs in rural and urban areas of the Coquimbo region of Chile and implications for disease transmission. Prev Vet Med.

[10] Downes M, Canty MJ, More SJ (2009). Demography of the pet dog and cat population on the island of Ireland and human factors influencing pet ownership. Prev Vet Med.

[11] Di Nardo A, Candeloro L, Budke CM, Slater MR (2007). Modeling the effect of sterilization rate on owned dog population size in central Italy. Prev Vet Med.

[12] Lund EM, Armstrong PJ, Kirk CA, Klausner JS (2006). Prevalence and risk factors for obesity in adult dogs from private US veterinary practices. Int J Appl Res Vet M.

[13] Kustritz MVR (2002). Early spay-neuter: clinical considerations. Clin Tech Small Anim Pract.

[14] Smith AN (2014). The role of neutering in cancer development. Vet. Clin. N. Am. Small Anim. Pract..

[15] de la Riva GT, Hart BL, Farver TB, Oberbauer AM, Messam LLM, Willits N, Hart LA (2013). Neutering dogs: effects on joint disorders and cancers in golden retrievers. PLoS One.

[16] Hart BL, Hart LA, Thigpen AP, Willits NH (2014). Long-term health effects of neutering dogs: comparison of labrador retrievers with golden retrievers. PLoS One.

[17] Salmeri K, Bloomberg M, Scruggs SL, Shille V (1991). Gonadectomy in immature dogs: effects on skeletal, physical, and behavioral development. J Am Vet Med Assoc.

[18] Hoffman JM, Creevy KE, Promislow DE (2013). Reproductive capability is associated with lifespan and cause of death in companion dogs. PLoS One.

[19] Shelton G, Schule A, Kass P (1997). Risk factors for acquired myasthenia gravis in dogs: 1,154 cases (1991–1995). J Am Vet Med Assoc.

[20] Shelton GD, Schule A, Kass PH (1998). Analysis of risk factors for acquired myasthenia in dogs. Ann N Y Acad Sci.

[21] Jacobs R, Murtaugh R, Crocker D (1984). Use of a microtiter Coombs’ test for study of age, gender, and breed distributions in immunohemolytic anemia of the dog. J Am Vet Med Assoc.

[22] Grossman CJ (1985). Interactions between the gonadal steroids and the immune system. Science.

[23] Tanriverdi F, Silveira L, MacColl G, Bouloux P (2003). The hypothalamic-pituitary-gonadal axis: immune function and autoimmunity. J. Endocrinol..

[24] Ahmed SA, Penhale W, Talal N (1985). Sex hormones, immune responses, and autoimmune diseases. Mechanisms of sex hormone action. Am J Pathol.

[25] Blount DG, Pritchard DI, Heaton PR (2005). Age-related alterations to immune parameters in Labrador retriever dogs. Vet Immunol Immunopathol.

[26] Ahmed SA, Talal N (1990). Sex hormones and the immune system—part 2. Animal data. Baillieres Clin Rheumatol.

[27] Greeley E, Kealy R, Ballam J, Lawler D, Segre M (1996). The influence of age on the canine immune system. Vet Immunol Immunopathol.

[28] Verthelyi D (2001). Sex hormones as immunomodulators in health and disease. Int Immunopharmacol.

[29] Wira CR, Rodriguez-Garcia M, Patel MV (2015). The role of sex hormones in immune protection of the female reproductive tract. Nat Rev Immunol.

[30] Bellumori TP, Famula TR, Bannasch DL, Belanger JM, Oberbauer AM (2013). Prevalence of inherited disorders among mixed-breed and purebred dogs: 27,254 cases (1995–2010). J Am Vet Med Assoc.

[31] Whitley N, Day M (2011). Immunomodulatory drugs and their application to the management of canine immune‐mediated disease. J. Small Anim. Pract..

[32] Pedersen NC (1999). A review of immunologic diseases of the dog. Vet Immunol Immunopathol.

[33] Nuttall T, Reece D, Roberts E (2014). Life-long diseases need life-long treatment: long-term safety of ciclosporin in canine atopic dermatitis. Veterinary record.

[34] Hughes A, Jokinen P, Bannasch D, Lohi H, Oberbauer A (2010). Association of a dog leukocyte antigen class II haplotype with hypoadrenocorticism in Nova Scotia Duck Tolling Retrievers. Tissue Antigens.

[35] Hughes AM, Bannasch DL, Kellett K, Oberbauer AM (2011). Examination of candidate genes for hypoadrenocorticism in Nova Scotia Duck Tolling Retrievers. Vet. J..

[36] Johnson KC, Mackin A (2012). Canine Immune-Mediated Polyarthritis: Part 2: Diagnosis and Treatment. J Am Anim Hosp Assoc.

[37] Craig M (2013). Disease facts: pemphigus foliaceus in the dog and cat. Companion Animal.

[38] Gordis L (2009). Epidemiology. 4th.

[39] Carpenter B, Gelman A, Hoffman M, Lee D, Goodrich B, Betancourt M, Brubaker MA, Guo J, Li P, Riddell A. Stan: a probabilistic programming language. J Stat Softw. 2015. 10.18637/jss.v076.i01 PMC9788645 36568334

[40] R Core Team. R: A language and environment for statistical computing. Vienna: R Foundation for Statistical Computing; 2015. http://www.R-project.org/.

[41] Viera AJ (2008). Odds ratios and risk ratios: what’s the difference and why does it matter?. South Med J.

[42] Gelman A, Carlin JB, Stern HS, Rubin DB (2014). Bayesian data analysis.

[43] Gelman A (2006). Prior distributions for variance parameters in hierarchical models (comment on article by Browne and Draper). Bayesian Anal.

[44] Gelman A, Rubin D (1992). Inference from iterative simulation using multiple sequences. Statistical Science.

[45] Hart BL, Hart LA, Thigpen AP, Willits NH (2016). Neutering of German Shepherd Dogs: associated joint disorders, cancers and urinary incontinence. J Vet Med Sci.

[46] Gleicher N, Barad DH (2007). Gender as risk factor for autoimmune diseases. J Autoimmun.

[47] Bourguignon E, Favarato ES, Guimarães LD, Ferreira TS. Dermatology in Dogs and Cats. Chapter 1, pp 3-34 in Insights from Veterinary Medicine, 2013. INTECH Open Access Publisher; http://dx.doi.org/10.5772/53660.

[48] Allenspach K, Wieland B, Gröne A, Gaschen F (2007). Chronic enteropathies in dogs: evaluation of risk factors for negative outcome. J Vet Intern Med.

[49] Shanley KJ (1985). Lupus erythematosus in small animals. Clin Dermatol.

[50] Rosenkrantz WS (2004). Pemphigus: current therapy. Vet Dermatol.

[51] Reimer ME, Troy GC, Warnick LD (1999). Immune-mediated hemolytic anemia: 70 cases (1988–1996). J Am Anim Hosp Assoc.

[52] Hanson J, Tengvall K, Bonnett B, Hedhammar Å (2015). Naturally occurring adrenocortical insufficiency–an epidemiological study based on a swedish‐insured dog population of 525,028 dogs. J Vet Intern Med.

[53] Frank LA, Rohrbach B, Bailey E, West J, Oliver JW (2003). Steroid hormone concentration profiles in healthy intact and neutered dogs before and after cosyntropin administration. Domest Anim Endocrinol.

[54] McClelland EE, Smith JM (2011). Gender specific differences in the immune response to infection. Arch Immunol Ther Exp (Warsz).

[55] Chiaroni-Clarke RC, Munro JE, Ellis JA (2016). Sex bias in paediatric autoimmune disease–Not just about sex hormones?. J Autoimmun.

[56] Arredouani MS (2014). New insights into androgenic immune regulation. OncoImmunology.

[57] Gaillard R, Spinedi E (1998). Sex-and stress-steroids interactions and the immune system: evidence for a neuroendocrine-immunological sexual dimorphism. Domest Anim Endocrinol.

[58] Hirokawa K, Utsuyama M, Kasai M, Kurashima C, Ishijima S, Zeng Y-X (1994). Understanding the mechanism of the age-change of thymic function to promote T cell differentiation. Immunol Lett.

[59] Utsuyama M, Hirokawa K, Mancini C, Brunelli R, Leter G, Doria G (1995). Differential effects of gonadectomy on thymic stromal cells in promoting T cell differentiation in mice. Mech Ageing Dev.

[60] Murakami M, Hosoi Y, Negishi T, Kamiya Y, Miyashita K, Yamada M, Iriuchijima T, Yokoo H, Yoshida I, Tsushima Y (1996). Thymic hyperplasia in patients with Graves’ disease. Identification of thyrotropin receptors in human thymus. J. Clin. Investig..

[61] Ahmed SA, Young P, Penhale W (1983). The effects of female sex steroids on the development of autoimmune thyroiditis in thymectomized and irradiated rats. Clin Exp Immunol.

[62] Krzyżewska-Młodawska A, Max A, Bartyzel BJ (2014). Influence of gonadectomy on serum ft4 concentrations in male and female dogs. Vet Med.

[63] Lee T-P, Chiang B-L (2012). Sex differences in spontaneous versus induced animal models of autoimmunity. Autoimmun Rev.

[64] Catchpole B, Ristic J, Fleeman L, Davison L (2005). Canine diabetes mellitus: can old dogs teach us new tricks?. Diabetologia.

[65] Pöppl A, Mottin T, González F (2013). Diabetes mellitus remission after resolution of inflammatory and progesterone-related conditions in bitches. Res Vet Sci.

[66] Nussinovitch U, Shoenfeld Y (2012). The role of gender and organ specific autoimmunity. Autoimmun Rev.

[67] Talbot NB, Butler AM, MacLachlan EA (1943). The effect of testosterone and allied compounds on the mineral, nitrogen, and carbohydrate metabolism of a girl with Addison’s disease. J Clin Investig.

[68] Ross I, Levitt N, Blom D, Haarburger D (2014). Male and female hypogonadism are highly prevalent in South Africans with Addison’s disease. Horm Metab Res.

[69] Lahita RG. The influence of sex hormones on the disease systemic lupus erythematosus. In: Springer seminars in immunopathology. 3 edn. Springer; 1986. p. 305-14. 10.1007/BF02099028 3544284

[70] Verdú EF, Deng Y, Bercik P, Collins SM (2002). Modulatory effects of estrogen in two murine models of experimental colitis. Am. J. Physiol. Gastrointest. Liver Physiol..

[71] Ghisletti S, Meda C, Maggi A, Vegeto E (2005). 17β-estradiol inhibits inflammatory gene expression by controlling NF-κB intracellular localization. Mol Cell Biol.

[72] Szczubial M, Kankofer M, Bochniarz M, Dąbrowski R (2015). Effects of ovariohysterectomy on oxidative stress markers in female dogs. Reprod Domest Anim.

[73] Nuttall T (2013). The genomics revolution: will canine atopic dermatitis be predictable and preventable?. Vet Dermatol.

[74] O’Neill DG, Church DB, McGreevy PD, Thomson PC, Brodbelt DC (2014). Prevalence of disorders recorded in dogs attending primary-care veterinary practices in England. PLoS One.

[75] Lian TM, Halliwell RE (1998). Allergen-specific IgE and IgGd antibodies in atopic and normal dogs. Vet Immunol Immunopathol.

[76] Halliwell R, DeBoer D (2001). The ACVD task force on canine atopic dermatitis (III): the role of antibodies in canine atopic dermatitis. Vet Immunol Immunopathol.

[77] Pucheu‐Haston CM, Bizikova P, Eisenschenk MN, Santoro D, Nuttall T, Marsella R (2015). Review: The role of antibodies, autoantigens and food allergens in canine atopic dermatitis. Vet Dermatol.

[78] Slattery ML, Potter JD, Curtin K, Edwards S, Ma K-N, Anderson K, Schaffer D, Samowitz WS (2001). Estrogens reduce and withdrawal of estrogens increase risk of microsatellite instability-positive colon cancer. Cancer Res.

[79] Lund E (2011). Epidemiology of canine atopic dermatitis. Vet Focus.

[80] Pedersen NC, Brucker L, Tessier NG, Liu H, Penedo MCT, Hughes S, Oberbauer A, Sacks B (2015). The effect of genetic bottlenecks and inbreeding on the incidence of two major autoimmune diseases in standard poodles, sebaceous adenitis and Addison’s disease. Canine Genet Epidemiol.

[81] Kustritz MVR (2014). Pros, cons, and techniques of pediatric neutering. Vet. Clin. N. Am. Small Anim. Pract..

[82] Casey RA, Loftus B, Bolster C, Richards GJ, Blackwell EJ (2014). Human directed aggression in domestic dogs (Canis familiaris): Occurrence in different contexts and risk factors. Appl. Anim. Behav. Sci..

[83] Spain CV, Scarlett JM, Houpt KA (2004). Long-term risks and benefits of early-age gonadectomy in dogs. J Am Vet Med Assoc.

